# Primary Spinal Dumbbell-Shaped Mesenchymal Chondrosarcoma: A Case Report and Review of the Literature

**DOI:** 10.7759/cureus.87386

**Published:** 2025-07-06

**Authors:** Homare Nakamura, Taigen Sase, Nobuyuki Yanagisawa, Masayuki Takagi, Hidetoshi Murata

**Affiliations:** 1 Department of Neurosurgery, St. Marianna University School of Medicine, Yokohama Seibu Hospital, Yokohama, JPN; 2 Department of Pathology, St. Marianna University School of Medicine, Kawasaki, JPN; 3 Department of Clinical Laboratory, National Hospital Organization Shizuoka Medical Center, Shizuoka, JPN; 4 Department of Neurosurgery, St. Marianna Medical University, Kawasaki, JPN

**Keywords:** chondrosarcoma, dumbbell-shape, dural repair, hey1::ncoa2, mesenchymal chondrosarcoma

## Abstract

We report a rare case of a dumbbell-shaped mesenchymal chondrosarcoma (MCS) in the lumbar canal. A 29-year-old man presented with lower back pain and pain in the left leg. Magnetic resonance imaging (MRI) showed a homogeneously enhanced dumbbell-shaped mass at the left L2-3 level. The mass was intradurally located and extended extradurally into the extraforaminal space through the left L2-3 intervertebral foramen. Computed tomography (CT) showed a calcified portion in the intradural mass. We exposed and excised the tumor via a posterior approach through a hemi-laminectomy of the left L2-3. The tumor had penetrated the dura mater and required repair. Following surgery, his symptoms resolved completely. The most likely histopathological diagnosis was MCS. Histologic examination of our surgical samples revealed the typical biphasic pattern, but there was also cartilage matrix resembling osteoid. Finally, molecular assays confirmed the presence of the HEY1::NCOA2 fusion gene.

Although spinal intradural extramedullary MCS is rare, only a few reports in the literature mention spinal dumbbell-shaped MCS. Following the removal of dumbbell-shaped MCS, the dura mater may require repair. While histopathological evaluation remains the gold standard for confirming a diagnosis of MCS, the HEY1::NCOA2 fusion gene is a specific molecular marker for MCS, and the presence of this gene has become a powerful tool for diagnosis.

## Introduction

Mesenchymal chondrosarcoma (MCS) is a rare and aggressive form of extraskeletal chondrosarcoma, accounting for less than 1% of all chondrosarcomas [1]. MCS can occur at any age; the peak age is in the second decade of life, which is younger than other malignant cartilage tumors [1]. MCS originates and affects the craniofacial bones, ribs, and spine, especially the thoracic spine [2-4]. Spinal MCS is exceedingly rare compared with intracranial lesions [5], and although intradural MCS are rare [1,2,5,6], dumbbell-shaped MCS are even rarer [4,7-12].

MCS shows a biphasic pattern of small cells with islands of atypical cartilage [1,2,5-10,12]. However, when histological evaluation and immunohistochemistry are inconclusive, diagnostic molecular tests to identify if any underlying fusion gene is present are essential for a definitive diagnosis of MCS [5].

In this report, we show that specific fusion genes are useful for diagnosis and provide key points for the surgical removal of dumbbell-type MCS, along with a literature review.

## Case presentation

A 29-year-old man presented with a chief complaint of lower back pain and pain in the left leg. He had no relevant past medical history. Conservative treatment, including administration of nonsteroidal anti-inflammatory drugs and use of an orthosis, had been tried by a local doctor in the preceding 12 months; however, symptoms had gradually worsened over four months. He was referred to our department for further treatment. On initial examination, he exhibited no loss of muscle power and no evidence of a sensory deficit in his lower extremities. Deep tendon reflexes, such as the patellar tendon reflex and Achilles tendon reflex, were hypoactive. The femoral nerve stretch test was positive in the left leg. No bladder or bowel disturbance was found.

**Figure 1:**
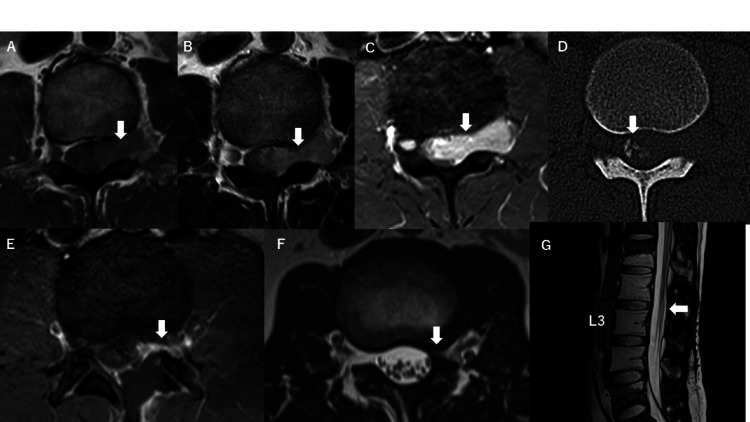
Preoperative and postoperative images. There is a dumbbell-shaped tumor with transforaminal extension at the left L2-3 level. It appears (A) homogeneously isointense (arrow) on the T1-weighted image, (B) slightly heterogeneously hyperintense (arrow) on the T2-weighted image, and (C) shows homogeneous enhancement (arrow) on gadolinium-diethylenetriamine pentaacetic acid (Gd-DTPA). (D) CT shows a calcified portion (arrow) in the tumor (D). (E) MRI (axial) showing the presence of residual tumor (arrow) after surgery on Gd-DTPA. (F, G) MRI showing no evidence of recurrence (arrow), five years after surgery on the T2-weighted image.

Magnetic resonance imaging (MRI) revealed a dumbbell-shaped tumor measuring 32 x 29 x 12 mm at the L2-3 level on the left side that showed homogeneously isointensity on T1-weighted images and slightly heterogeneously hyperintensity on T2-weighted images and was homogeneously enhanced with Gd-DTPA (Figures 1A-1C). The tumor extended from the left side of the L2-3 spinal canal through the intervertebral foramen. Plain and reconstructive computed tomography (CT) images showed a calcified mass within the dura mater at the L2-3 levels (Figure 1D). The differential diagnoses were schwannoma and meningioma.

**Figure 2:**
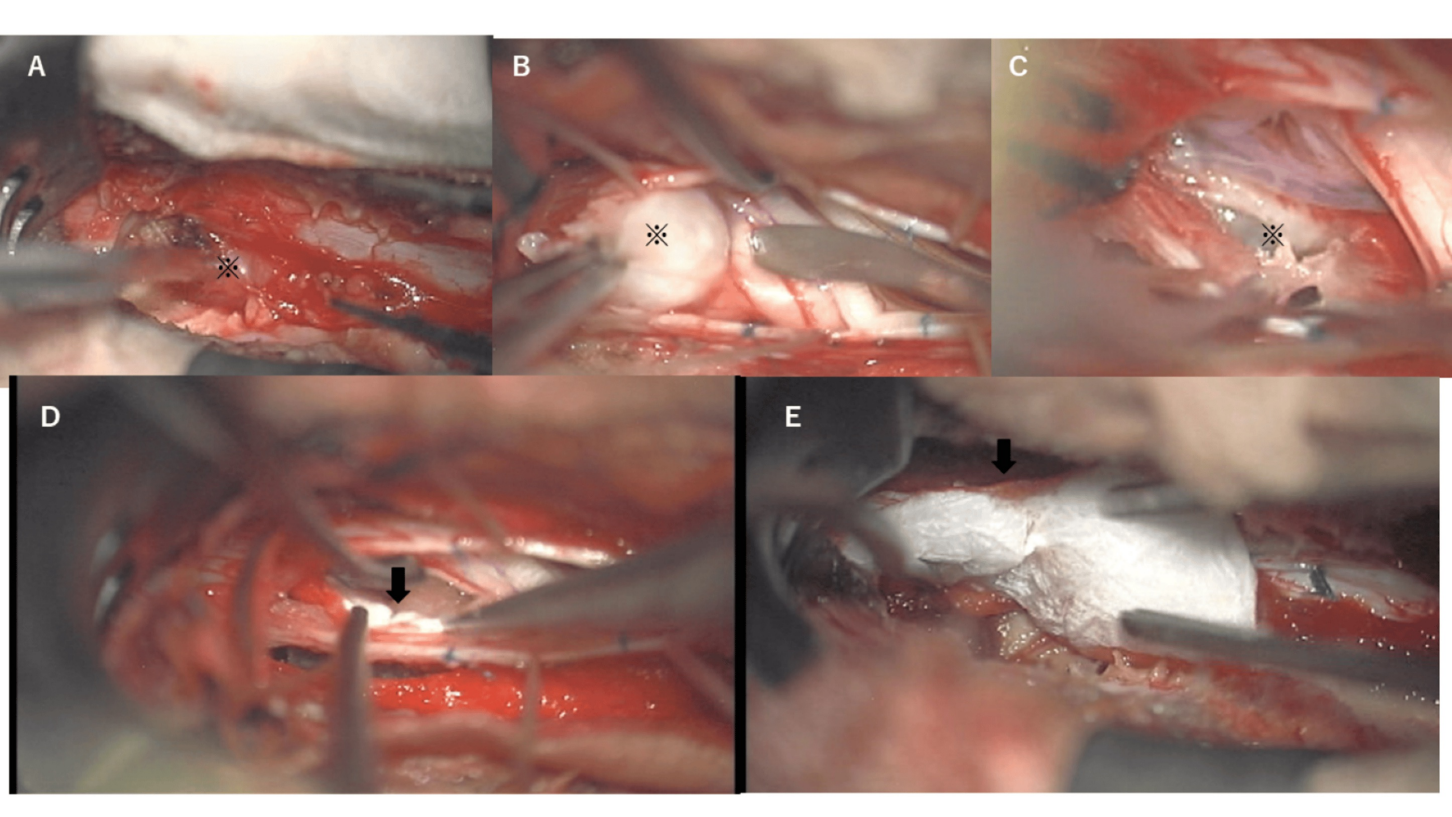
Intraoperative photographs. (A) The fibrous capsule covering the surface of the extradural tumor (※). The vascular-rich, fragile component appeared inside. (B) Intraoperative image showing that the intradural tumor (※) was attached to the dura and did not involve the nerve roots of the cauda equina. (C) A penetration (※) was identified at the left lateral wall of the thecal sac after removal of the intradural mass. (D) A collagen matrix graft was inserted into the subdural space as an inlay graft (arrow) and placed on the inner side of the dural defect. (E) An onlay graft was then placed over the dural defect, covering it medially and externally.

Surgery was performed under general anesthesia in the prone position with intraoperative neuromonitoring. A left-sided lateral laminectomy was performed at L2 and L3 to expose the posterior surface of the thecal sac. First, an extradural mass was located at the left lateral aspect of the thecal dural sac (Figure 2A), the epidural space just above the left L3 nerve root. After debulking the extradural mass using an ultrasonic surgical aspirator, we performed subcapsular removal of the extradural mass through the intervertebral foramen. Next, the dura mater was incised just above the posterior surface of the thecal sac. A white solid mass was observed, penetrating the left dura mater (Figure 2B). The mass was dissected around the cauda equina. After removal of the mass, the left lateral wall of the thecal sac was also found to have been penetrated (Figure 2C). Collagen matrix grafts (DuraGen; Integra LifeSciences, Princeton, NJ) were cut to be larger than the dural defect. The collagen matrix graft was inserted into the subdural space as an inlay graft (Figure 2D). An onlay graft was then placed over the dural defect (Figure 2E), covering it medially and externally, hydrated with saline, and reinforced with fibrin glue. The collagen matrix graft was inserted into the subdural space as an inlay graft (Figure 2D). Then, an onlay graft was placed into the dural defect (Figure 2E), covering the defect medially and externally, hydrated with saline, and reinforced with fibrin glue. We completed the surgery after closing a dural incision in the posterior wall.

**Figure 3:**
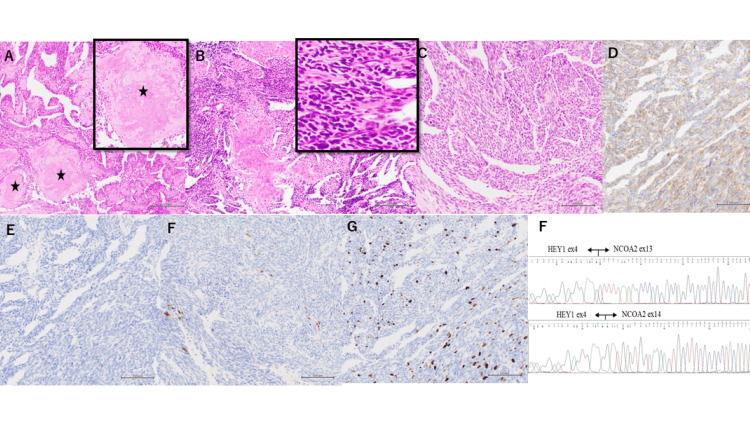
Histological examination and RT-PCR. (A and B) (×100) Histological examination revealed that the tumor was hypercellular and composed of undifferentiated small round and spindle cells with a high nucleocytoplasmic ratio, along with dispersed cartilage islands. However, cartilage islands with calcification resemble bone formation (black asterisks). The black frame indicates high magnification. (C) (×200) The small round cells were arranged in structures separated by prominent staghorn-shaped blood vessels. (D-F) (×200) Immunohistochemical staining revealed that the tumor cells were positive for CD99 (D) and negative for epithelial membrane antigen (EMA) (E) and S-100 protein (F). (G) (×200) The Ki-67 labeling index was approximately 10%-20%. (H) RT-PCR confirmed the presence of HEY1::NOCOA fusion. EX4-EX13 and EX4-EX14 fusion genes were detected. RT-PCR, reverse transcription polymerase chain reaction

Histological examination showed the tumor was hypercellular and composed of undifferentiated small round and spindle cells with a high nucleocytoplasmic ratio, and dispersed cartilage islands. However, cartilage islands with calcification resemble bone formation (Figures 3A-3B). The small round cells were arranged in structures separated by prominent staghorn-shaped blood vessels (Figure 3C). For immunohistochemistry, the tumor cells were positive for CD99 and negative for epithelial membrane antigen (EMA) and S-100 protein (Figures 3D-3F). The Ki-67 labeling index was approximately 10%-20% (Figure 3G). They lacked typical histopathological features, and no diagnosis was reached. Therefore, a genetic search was performed. The HEY1::NCOA2 fusion transcript was detected by reverse transcription polymerase chain reaction (RT-PCR). (Figure 3H). Based on these findings, the patient was diagnosed with MCS.

Radiologic imaging confirmed that the mass had been subtotally resected (Figure 1E). After surgical intervention, he experienced mild lower back pain when moving sometimes, and the pain in his left leg had disappeared. No cerebrospinal fluid leakage after surgery was observed. Based on the histological and molecular biological analyses described above, a final diagnosis of MCS was made. Therefore, he received adjuvant radiotherapy with 66 Gy in 33 fractions. He was followed up for five years without any recurrences, based on neuroimaging studies that were conducted without enhancement due to the presence of allergies (Figures 1F, 1G).

## Discussion

In 1959, Lichtenstein and Bernstein first described MCS. MCS represents a rare subtype of chondrosarcoma [13]. Although chondrocytes are thought to be the cells of origin, chondrosarcomas are occasionally found in extraskeletal tissues [8]. The exact histogenesis of intradural chondrosarcomas remains obscure [9,11]. Extraskeletal MCS accounts for 33%-50% of total MCSs [6]. The intradural extramedullary location of MCS is rare, with a limited number of documented cases [1,2,5,6]. Piscopo et al. reviewed 16 MCSs in the primary intradural MCS [5] and found that dumbbell-shaped MCSs are exceedingly rare (Table 1) [4,7-12]. In this dumbbell-shaped MCS, there are cases where MCS is present inside and outside the dura mater, and surgery may require repair of the defect where the tumor has penetrated the dura mater, depending on the degree of dural defect, for the prevention of cerebrospinal fluid leakage. In this case, the dural defect was large; therefore, dural repair was performed.

**Table 1:** Summary of primary dumbbell-shaped mesenchymal chondrosarcoma. M, male; F, female; NA, not available; GTR, gross total resection; STR, subtotal resection

Author (year)	Age (years) (sex)	Location	Treatment	Dural repair (material)	Adjuvant therapy	Recurrence	Outcome (duration)
Chan et al. (1984) [7]	10 (F)	T3-4 extradural	NA	No	Radiation therapy and chemotherapy	No	Alive, (18 months)
Reif et al. (1987) [4]	3 (M)	L1-5 extradural	NA	No	Radiation therapy and chemotherapy	Yes (brain metastasis)	Died (3 months after first operation)
Lorenzo et al. (1989) [8]	40 (F)	L5-S1 extradural	GTR	No	Radiation therapy and chemotherapy	No	Alive (5 years)
Bae et al. (2011) [9]	44 (M)	T7 extra and intradural	GTR	Yes (primary suture)	Radiation therapy and chemotherapy	No	Alive (2 years)
Iida et al. (2014) [11]	10 (F)	L4 extra and intradural	GTR	Yes (GORE-TEX)	No	No	Alive (3 years)
Chen et al. (2016) [10]	26 (F)	L3-5 extradural	NA	No	Radiation therapy	Yes (local recurrence)	NA
Chen et al. (2022) [12]	19 (M)	T12-L2 extra and intradural	GTR	NA	No	No	Alive (1 year)
Our case	29 (M)	L2-3 extra and intradural	STR	Yes (Duragen)	Radiation therapy	No	Alive (5 years)

In our case, radiographical findings showed a dumbbell-shaped tumor with calcification through the intervertebral foramen. A schwannoma commonly appears as a spinal dumbbell tumor [14], with approximately 10%-15% of schwannomas having this shape [15]. In contrast, calcification in spinal tumors is most commonly associated with meningiomas. Although it is well known that calcification can occur in meningiomas, the reported frequency is only 1%-5% of all cases [16]. Schwannoma and meningioma are the two most common intraspinal tumors. We consider the dumbbell-shaped tumor to be an atypical lesion. We initially assumed the present patient’s lumbar tumor was a schwannoma or meningioma. However, it was MCS, which neurosurgeons are not familiar with. Calcification and enhancement could be a characteristic finding of MCS, but imaging examinations to identify MCS have been unreliable. Although preoperative diagnosis of spinal MCS is difficult, MCS should be considered in the differential diagnosis of an atypical calcified tumor identified on enhanced MRI.

Histopathological evaluation is the gold standard for confirming the diagnosis of MCS, and a novel specific fusion gene, HEY1::NCOA2, was discovered in 2012 [17]. HEY1::NCOA2 fusion is a specific molecular marker for MCS, and it has become a powerful tool for diagnosis. HEY1::NCOA2 served as a useful diagnostic molecular signature, especially in cases where surgical samples lacked typical pathological features [18]. In our case, HEY1::NCOA2 gene fusion helped to confirm the diagnosis of MCS.

 Finally, the overall 10-year survival rate for primary spinal MCS is estimated to range from 21% to 67% [2,4,5,8]. Total surgical resection has proven essential for good outcomes, and the role of adjuvant radiotherapy and chemotherapy remains controversial. Because it was a subtotal resection, he underwent radiation therapy [2,5,10,11]. Long-term follow-up is necessary.

## Conclusions

Dumbbell-shaped MCSs are exceedingly rare, and their removal may require dural repair. HEY1::NCOA2 fusion is a specific molecular marker for MCS. HEY1::NCOA2 served as a useful diagnostic molecular signature, especially in cases where surgical samples lacked typical pathological features.
